# Experiences and Support Needs of Siblings of Individuals With Prader‐Willi Syndrome: An Integrative Systematic Review

**DOI:** 10.1111/jar.70171

**Published:** 2026-01-11

**Authors:** Meghana Wadnerkar Kamble, Jen Dawe, Karen Bunning

**Affiliations:** ^1^ School of Health Sciences, Faculty of Medicine and Health, University of East Anglia Norwich UK

**Keywords:** experiences, family, parents, Prader‐Willi syndrome (PWS), siblings

## Abstract

**Background:**

Prader‐Willi syndrome is a complex neurogenetic condition. Recognised to affect the entire family, little is known of the sibling experience.

**Objectives:**

Review the experiences and support needs of siblings of people with Prader‐Willi syndrome.

**Methods:**

Eight databases, registers and grey literature were searched covering October 2000–May 2024. Search terms were based on siblings and their experiences of a sibling who has Prader‐Willi syndrome. Quality appraisal deployed the Mixed Methods Appraisal Tool. Qualitative findings were assessed using thematic analysis. Quantitative results were summarised and integrated using narrative synthesis.

**Results:**

Out of 7588 results, seven studies were selected. Quantitative reports primarily highlighted negative psychological effects, whilst qualitative reports concentrated on family relations. Narrative synthesis identified psychological impact, influence on family relationships, and the effect of familial characteristics.

**Conclusions and Implications:**

Sibling experiences are shaped by family context. The need for family‐centred approach in research and practise is advocated.

## Lay Summary

- Family context shapes sibling experiences in Prader‐Willi syndrome.
- Evidence on sibling experiences is limited, lacks qualitative depth, and is currently not widely generalisable.
- Understanding the family system can have implications for the agency or lack of agency felt by the person with Prader‐Willi syndrome.
- Future research can use exploratory qualitative design to capture the family's voice and seek multiple perspectives including from the person with Prader‐Willi syndrome.

## 1 Background

Caring for a child with intellectual and developmental disabilities can impact the entire family (Brekke and Alecu 2023; Masefield et al. 2020; Totsika et al. 2011). For siblings, having a brother or sister with a disability can shape their personal and social development (Lamsal and Ungar 2019; Caliendo et al. 2020). However, research on siblings of children with disabilities has often relied on parental reports (Guite et al. 2004), with limited input from the siblings themselves. When siblings' perspectives are included, discrepancies often emerge between their views and those of their parents. For instance, Guite et al. (2004) found that parents reported higher levels of adjustment problems than the siblings did. Studies that do capture siblings' experiences, either directly or indirectly, also show varied results (e.g., Cuskelly and Gunn 2003). The experience of having a brother or sister with a disability is often quite complex. Siblings can have a negative experience, leading to behavioural challenges (Moyson and Roeyers 2011), or a positive experience, fostering personal growth and resilience (Opperman and Alant 2003). Thus, the sibling experience likely encompasses both positive and negative aspects. However, sibling research has often focused on negative aspects (Hastings 2016), with limited exploration of their lived experiences (Hanvey et al. 2022). Siblings frequently support their disabled family member, sometimes taking on primary caregiver roles, which can affect family dynamics (Coyle et al. 2014).

The type of disability can greatly influence its impact on siblings. For example, having a sibling with Down syndrome can foster empathy in well siblings (Cuskelly and Gunn 2003). Siblings of individuals with autism may experience a range of emotions, including social isolation (Benderix and Sivberg 2007). Rare syndromes add further complexity to the sibling experience due to condition‐specific traits. This is evident in Williams syndrome, a rare genetic disorder affecting 1 in 7500–10,000 people (WSA 2025). Siblings of individuals with Williams syndrome report a close bond characterised by both affection and frustration due to the syndrome's unique traits (Cebula et al. 2025). There is growing recognition of the need to understand caregiver burden and sibling needs in neuronopathic Hunter syndrome (Eisengart et al. 2020; Grant 2018), another rare genetic condition affecting 1 in 150,000 male births (Project Alive 2025). Most rare diseases have a genetic cause and a low prevalence rate. Over 7000 types of rare diseases affect approximately 300 million people worldwide (The Lancet Global Health 2024). Rare diseases pose interconnected challenges of limited awareness and support, marginalising affected individuals and their families. Consequently, sibling experiences and voices often go unheard in the context of rare diseases. Understanding siblings' experiences in specific or rare syndromes, such as Prader‐Willi syndrome, is crucial to addressing family support needs (Marquis et al. 2023).

### 1.1 Prader‐Willi Syndrome

Prader‐Willi syndrome is a rare, complex, multi‐system neurodevelopmental disorder primarily caused by the absence of paternally expressed genes on chromosome 15q11‐q13 (Angulo et al. 2015; Cassidy et al. 2012). It affects 1 in 10,000 to 30,000 individuals across all races, ethnicities, and genders equally (Driscoll et al. 2023). Those with Prader‐Willi syndrome often experience developmental delays and mild to moderate intellectual disabilities (Driscoll et al. 2023). The syndrome is characterised by unique traits such as lifelong hyperphagia (excessive and insatiable hunger), food obsession, and related physical conditions like obesity (Hedgeman et al. 2017). These traits may compel families to adopt extreme measures, such as locking fridges, restricting kitchen access, and eating separately (Currie et al. 2024).

Additionally, Prader‐Willi syndrome can be associated with behavioural phenotypes including temper tantrums, anxiety, skin‐picking, obsessive‐compulsive behaviours, and difficulties with executive functions and social cognition (Schwartz et al. 2021; Sinnema et al. 2011). There is also a risk of comorbid conditions such as psychosis (Larson et al. 2014). The presence of these multi‐systemic traits can pose significant challenges for caregivers, affecting their mental health and overall quality of life (Kayadjanian et al. 2018). However, there is limited research on the broader impacts of Prader‐Willi syndrome within the family system, particularly regarding the experiences of siblings.

### 1.2 Voluntary Sectors

Voluntary sector organisations highlight the necessity for further research into the experiences of siblings of individuals with developmental disabilities (Hastings 2014). This need is supported by the UK Government's Child and Families Act (2014), which underscores the importance of considering family relationships when one or more members have a disability. Additionally, charities focused on disabilities, including developmental disorders, stress the importance of family support (e.g., PWSA UK 2022; IPWSO 2023; DSA 2021), addressing impacts on family finances, wellbeing, and caregiving demands (e.g., Mencap 2022; PWSA USA 2023; DSA 2021). Whilst practical advice for families is essential, the focus has predominantly been on the family unit through the parental perspective, rather than addressing the support needs of siblings. This approach contrasts with guidance from organisations like the National Autistic Society (2022), which asserts that siblings have distinct needs and are best positioned to identify them. Voluntary sector organisations, such as for Prader‐Willi syndrome, emphasise the support needs of relatives (PWSA UK 2022; IPWSO 2023), though there is limited evidence supporting well‐defined interventions.

### 1.3 Gap in Evidence

The lack of interventions for siblings of individuals with developmental disabilities has been noted by authors like Hayden et al. (2019), who suggest implementing a school‐based intervention for younger siblings. Consequently, research on siblings' experiences lags behind studies on parent–child relationships (McHale et al. 2013), overlooking a crucial subsystem within the family (Bowen 1978). This gap is particularly evident in the context of Prader‐Willi syndrome, which has unique characteristics that underscore the need for sibling‐focused research (PWSA UK 2022; IPWSO 2023). Addressing this gap is essential to understand how the unique challenges of having a family member with Prader‐Willi syndrome affect sibling and family dynamics. In March 2022, the authors reviewed the literature on Prader‐Willi syndrome to explore sibling perceptions. This review identified several studies on autism and learning disabilities, but only one small‐scale mixed methods study (Mazaheri et al. 2012) specifically addressed the sibling perspective in Prader‐Willi syndrome. This initial scoping exercise informed the current review, which aims to explore the experiences and support needs of siblings of individuals with Prader‐Willi syndrome from both the siblings' and parents'/carers' perspectives.

## 2 Aims and Review Questions

This integrative review aimed to establish what is known about the experiences and support needs of siblings of individuals with Prader‐Willi syndrome from the sibling's own point of view and the parent/carer's point of view to explore how the sibling view is captured across the family. An integrative review was chosen to get a better insight into the context and the factors that are contributing towards the sibling's experiences. This led to two inter‐linked review questions:

1. What are the experiences and support needs of siblings who have a brother or sister with Prader‐Willi syndrome from the sibling perspective?
2. How do parents perceive the siblings' experience of having a brother or sister with Prader‐Willi syndrome in the family?

## 3 Materials and Methods

This systematic review was conducted using the Preferred Reporting Items for Systematic Reviews and Meta‐Analyses (PRISMA 2020) guidelines (Page et al. 2021). The protocol of this systematic review was informed by the pilot scoping exercise. The review protocol was subsequently published on PROSPERO (Wadnerkar Kamble et al. 2022, registration number CRD42022366918).

## 4 Search

A systematic search was conducted in November 2022 and updated in June 2024 to ensure currency of records. Eight databases associated with health and social care research were searched, namely: COCHRANE central register, PubMed, Embase, CINAHL, PsychINFO, Web of Science, Scopus and EBSCO. Search terms were developed using the PEO framework (population, exposure, outcomes), which lends itself to cover quantitative and qualitative study designs. Search terms were based on the population of interest (P), i.e., siblings of individuals with Prader‐Willi syndrome and parents or carers; exposure (E), i.e., the experience of having a sibling with Prader‐Willi syndrome; and outcomes (O), i.e., primary outcomes around the experiences or perceptions of the siblings; secondary outcomes around wellbeing, relational functioning and quality of life. Truncations were used for word inflexion. Sources of grey literature were also searched and included, i.e., Cochrane library, PWSA UK, EThOS, Advanced Google Search, OpenGrey, WHO Iris, Google Scholar and CORE. Reference lists of relevant studies shortlisted during the searches were also investigated Supporting Information A. Table 1 details the search terms.

**TABLE 1:** Search terms.

Population	Experience	Outcomes
Sibling* OR brother* OR sister* OR parent* OR carer* OR caregiver* OR grandparent* OR adopt* OR foster* OR ‘local authorit*’ OR ‘siblings of children with disabilit*’	‘Prader‐Willi syndrome’ OR PWS OR ‘prader‐willi’ OR ‘neurodevelopmental condition*’ OR ‘developmental disabilit*’	Support* OR help* OR experience* OR perception* OR attitude* OR view* OR feeling* OR emotion* OR affect*

### 4.1 Inclusion Criteria

Papers in peer‐reviewed journals and grey literature, written in English, published between October 2000–May 2024 were considered eligible for inclusion. The period of 24 years was considered in order to include seminal articles on the review topic that were published during the early 2000s. Papers and reports were included if the study sample comprised sibling(s) (of any age) of a person (of any age) who has Prader‐Willi syndrome and/or parents/carers of at least one child who has Prader‐Willi syndrome, and at least one child who does not have Prader‐Willi syndrome. Studies had to be on any aspect of sibling experience and/or support needs as reported by the siblings and/or parents. Additionally, all study types (i.e., quantitative, qualitative, or mixed methods), validated and non‐validated outcome measures on the primary and secondary outcomes, and any outcomes on the siblings' experience and or support needs were also included. Outcomes were included only when they were reported either by the siblings themselves and/or by their parent/carer. Studies that included Prader‐Willi syndrome within a broader spectrum of intellectual and developmental disabilities were considered, provided that specific information about Prader‐Willi syndrome could be isolated. Studies were excluded if the siblings were not siblings of a person with Prader‐Willi syndrome or if the study concerned only family members or caregivers with no information on the well sibling or if there were no independent data points relating to Prader‐Willi syndrome.

The screening process was initially piloted by the first (MWK) and second (JD) authors using one database, achieving an 80% agreement rate to ensure adherence to the inclusion criteria. Subsequently, the second author (JD) independently screened all titles and abstracts. Sources that met all inclusion criteria were retrieved in full text and independently screened by the raters. Any disagreements regarding inclusion were resolved through discussion between the first and second authors. If consensus was not reached, the third author (KB) was consulted. There was good agreement between the two raters for the full‐text screening, *κ* = 0.737, *p* = 0.016.

### 4.2 Assessment of Risk of Bias in Included Studies

The Mixed Methods Appraisal Tool (MMAT, version 2018, Hong, Fàbregues, et al. 2018), which is a widely used tool (Guetterman et al. 2022) with proven psychometric properties (Hong, Gonzalez‐Reyes, and Pluye 2018; Hong et al. 2019; Souto et al. 2015), was used to assess quality and risk of bias. Each of the five screening questions on the MMAT is weighted 20% for a ‘yes’ and 0% for ‘no.’ Studies were included when the quality was scored at 80% or above. At least 30% of potential discrepancies in the findings were discussed with the 1st author (MWK) until consensus was reached.

### 4.3 Data Extraction

A data extraction table was developed in Excel, based on the Cochrane Consumers and Communication Review Group Data Extraction Template (2022) and piloted by the 1st author (MWK). The 2nd author (JD) independently extracted the data from the included studies. Data were extracted for research question, study design, recruitment, study participants, outcome measures, intervention details where applicable, and findings.

### 4.4 Data Analysis

Thomas and Harden's (2008) recommendations for conducting thematic analysis were used to synthesise the qualitative findings. The main codes were identified from the results/findings and discussion sections of the studies included. Per best practise for thematic analysis, the systematic coding of the texts was developed iteratively. Two authors (JD and MWK) discussed the first iteration of codes and their correspondence to the review questions. When relevant, codes were re‐organised and renamed. Codes were condensed to form themes. Any discrepancies or disagreements, including their labelling and organisation, were resolved by discussion with the third senior author (KB). The final iteration of themes was completed once the two authors (JD and MWK) agreed that the themes wholly answered the review questions, i.e., all codes had been adequately captured, no extraneous findings had been included, and that hierarchical organisation and labelling of themes was complete and resonant Supporting Information B. Heterogeneity of the quantitative studies negated the use of a meta‐analysis. Instead, statistically significant results were captured from the quantitative findings, and summarised and integrated with qualitative findings for the narrative synthesis.

## 5 Results

**FIGURE 1:**
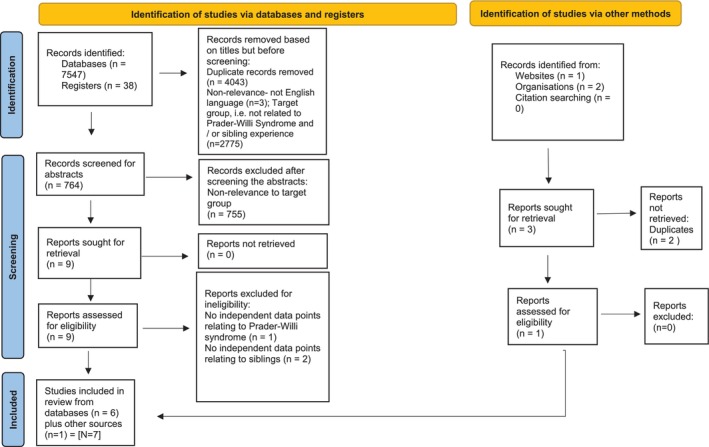
PRISMA flow chart.

Search across the eight databases resulted in 7547 retrievals, and 38 from registers totaling to 7585 (Figure 1). Analysis in Excel for study titles led to 6821 records being removed (duplicates = 4043; not in the English language = 3; and irrelevant to the target group = 2775). This left 764 records for subsequent screening of abstracts, which identified 755 records that did not meet the inclusion criteria and thus were removed. This resulted in six records from databases and registers. A further three records were found from other sources, which had two duplicate records and thus were removed, leaving one record. This led to a final sample of seven studies from the initial 7588 records. These seven studies comprised one qualitative study, three quantitative studies, and three mixed‐methods studies. Amongst the mixed‐methods studies, one was an Honours thesis and another was a Doctoral thesis.

### 5.1 Study Characteristics

The term ‘sibling’ refers to typically functioning brothers and sisters, whilst ‘sibling with Prader‐Willi syndrome’ specifies the primary condition. Across seven studies, the distribution of reports from parents and siblings was as follows: four studies included both parent and sibling perspectives (Allen 2011; Bennett Murphy et al. 2022; Mazaheri et al. 2012; Rae‐Seebach 2008), two studies included only sibling reports (O'Neill and Murray 2016; French 2022), and one study had parents reporting on behalf of siblings (Meade et al. 2020). Six studies focused exclusively on Prader‐Willi syndrome, except for O'Neill and Murray (2016), which included Prader‐Willi syndrome amongst other developmental disabilities. Additionally, one study explored the views of young people with Prader‐Willi syndrome (Allen 2011), and another used a comparator sample of siblings of neurotypical individuals (French 2022).

#### 5.1.1 Sample and Demographics

Three studies were based in the USA (Bennett Murphy et al. 2022; Mazaheri et al. 2012; Rae‐Seebach 2008); two in England (Allen 2011; O'Neill and Murray 2016); one each in Ireland (Meade et al. 2020) and Canada (French 2022). The final sample of seven studies involved a total of 673 participants (80 in qualitative studies; 593 in quantitative studies). Of the total participant number, 490 (72.80%) of the participants were siblings. In the siblings' sample, there were 164 (33.46%) siblings of people with Prader‐Willi syndrome. Of the total participant number, parents or carers made up 163 of the sample (who were not siblings) (24.21%). These were all parents of people with Prader‐Willi syndrome. Twenty of the total participants were people with Prader‐Willi syndrome (Table 2). It was not possible to establish whether some participants had participated in more than one study. For instance, the Rae‐Seebach (2008) and Mazaheri et al. (2012) studies drew from the same sample, with the Mazaheri et al. (2012) reporting on a sub‐set of measures with a different focus as compared to the Rae‐Seebach (2008). For this reason, the Rae‐Seebach (2008) and Mazaheri et al. (2012) studies were treated as separate studies.

**TABLE 2:** Demographics from included studies.

Study	Sample (*N*, age and setting)	Demographics (sex, ethnicity)
Quantitative studies		
Bennett Murphy et al. 2022	Parents and young/young adult siblings in a Prader‐Willi syndrome focused study	None reported
	*N* = 144. 86 parents. 58 siblings (10–18 years) of people with Prader‐Willi syndrome. Ages of people with Prader‐Willi syndrome not reported. Recruited: Prader‐Willi syndrome support organisations.	
Meade et al. 2020	Parents reporting on siblings, with physical measurements taken for children/adolescents with Prader‐Willi syndrome in a Prader‐Willi syndrome focused study	89% mothers, 11% fathers.
	*N* = 19. 19 parents; of these 5 reported on siblings. Children with Prader‐Willi syndrome (median age 7.9 years) and parents/carers (age not reported) of children with Prader‐Willi syndrome.	Children with Prader‐Willi: 74% female, 26% male
	Recruited: Children's Health Ireland.	Ethnicity: not reported
O'Neill and Murray 2016	Adult siblings in a study on developmental disabilities	Siblings of people with Prader‐Willi: 34 males, 98 females.
	*N* = 132. Adult siblings (aged 19–71 years) of a sibling with a developmental disability, incl 26 (mean age 31 years) who had a sibling with Prader‐Willi syndrome (mean age 29 years). Recruited: UK disability charities.	Ethnicity: not reported
Qualitative studies		
Allen 2011	Parents, siblings and young people with Prader‐Willi syndrome	None reported
	*N* = 80. 34 parents, 26 siblings ages not known. 20 young people with Prader‐Willi syndrome (ages 11–15 years) living with one/both parents, were an only child or child with siblings. Recruited: Prader‐Willi syndrome support organisation.	
Mixed methods studies		
Rae‐Seebach 2008 (Doctoral Thesis)	Mothers and young/young adult siblings in a Prader‐Willi syndrome focused study	Siblings: 61.5% female, 38.5% males
	*N* = 25. 12 mothers (average age 45 years), 13 siblings (mean age 16 years). Siblings with Prader‐Willi syndrome mean age 1 month‐12 years. Recruited: Prader‐Willi syndrome associations and the Genetic Medicine Central California Prader‐Willi syndrome clinic in California.	Ethnicity siblings: 83.3% Caucasian, Latino 8.3%, Asian 8.3%.
French 2022 (Honours thesis)	Young adult/adult Siblings in a Prader‐Willi syndrome and comparator study	None reported
	*N* = 248. 28 siblings of people with Prader‐Willi syndrome and 220 siblings of people who did not have Prader‐Willi syndrome. All participants aged 16 or above. Recruited: Prader‐Willi syndrome support organisation and social media.	
Mazaheri et al. 2012	Mothers and young/young adult siblings in a Prader‐Willi syndrome focused study	All mothers, Siblings: 8 females, 5 males.
	*N* = 25. 13 siblings (aged 12–19 years) of individuals with Prader‐Willi, 12 mothers (aged 30–55 years) of children with Prader‐Willi syndrome with at least one other child who did not have Prader‐Willi syndrome. Recruited: Prader‐Willi syndrome associations and the Genetic Medicine Central California Prader‐Willi syndrome clinic in California.	Ethnicity mothers: Caucasian 83.3%, Latino 8.3% and Asian 8.3%
		Ethnicity siblings: Caucasian 81.8%, Latino 9.1%, Asian 9.1%

The overall sample was homogenous with a restricted age spread (Table 2). Although it was difficult to ascertain the mean age of participants as age was variously detailed, i.e., either as age range or exact age. Four studies considered younger siblings from aged 10 onwards (Allen 2011 ages not known; Bennett Murphy et al. 2022, 10–18 years; Mazaheri et al. 2012, 12–19 years; Rae‐Seebach 2008 mean age 16 years). Where specified, most participants were female, i.e., mothers and sisters of people with Prader‐Willi syndrome. Participant characteristics, such as ethnicity, socioeconomic status, and family composition, were reported inconsistently across the studies. Two studies (Mazaheri et al. 2012; Rae‐Seebach 2008) reported on ethnicities of participants. These had 82.75% Caucasian parents and 82.55% siblings, 8.3% Latino and Asian parents, and 8.7% Latino and Asian siblings (Table 2).

#### 5.1.2 Study Design and Outcome Measures

One study employed qualitative methods (Allen 2011). Three studies utilised quantitative methodologies (Bennett Murphy et al. 2022; Meade et al. 2020; O'Neill and Murray 2016). Three studies adopted mixed methods (French 2022; Mazaheri et al. 2012; Rae‐Seebach 2008). This results in a total of six reports using quantitative methods and four reports using qualitative methods. The qualitative methods in the mixed‐methods studies included semi‐structured interviews with siblings (Rae‐Seebach 2008), and with both siblings and mothers (Mazaheri et al. 2012). French (2022) incorporated qualitative questions at the end of each series of quantitative surveys.

Self‐report standardised norm and criterion‐referenced questionnaires were commonly utilised in six out of the seven studies. These questionnaires assessed the experience of having a family member with Prader‐Willi syndrome, covering various aspects such as: caregiver burden (French 2022; Meade et al. 2020); quality of life (Meade et al. 2020; Mazaheri et al. 2012); stress (Bennett Murphy et al. 2022; French 2022; Rae‐Seebach 2008; Mazaheri et al. 2012); well‐being (Bennett Murphy et al. 2022; French 2022; Mazaheri et al. 2012; O'Neill and Murray 2016); family functioning and attachment (Rae‐Seebach 2008; Bennett Murphy et al. 2022); and affect (Bennett Murphy et al. 2022). Meade et al. (2020) developed a bespoke questionnaire from a subset of a broader sibling questionnaire to capture activities undertaken by siblings with developmental disorders, sibling relationships, and well‐being. Additionally, one study (Meade et al. 2020) included physical measurements (height/weight) of the child with Prader‐Willi syndrome. The mixed‐method studies also incorporated qualitative questionnaires (French 2022; Mazaheri et al. 2012; Rae‐Seebach 2008). Allen (2011) captured the family's voice to understand management of dietary issues and everyday practises.

#### 5.1.3 Theoretical Frameworks

Two of the seven studies explicitly identified a grounding theoretical framework, i.e., a sociological embodied epistemology (Allen 2011) and attachment theory (Rae‐Seebach 2008). See Table 3 for a summary of included studies.

**TABLE 3:** Summary of included studies.

Study; location; duration	Funding	Study quality score %	Aim	Study design, theory framework	Siblings related key findings
Sibling and parent reports					
Bennett Murphy et al. 2022; USA; Not reported	US Department of Health and Human Services	80% (sampling strategy unclear in relation to the research question)	Psychological distress and symptoms of PTSD in siblings.	Quantitative online survey for affect, wellbeing and family using standardised measures analysed using regression analyses.	*Siblings:* 58.9% of siblings described PTSD symptoms exceeding clinical cut‐off. PTSD symptoms were associated with greater frequency of reported negative affect (*p* < 0.001), but unrelated to resilience, positive affect, or the continuous positivity ratio.
				*Sibling questionnaires:* CPSS, PANAS‐C, CD‐RISC 10	*Parents:* 38.67% of siblings had clinically significant psychological distress. Psychological distress was related to level of family organisation (*p* = 0.013) and control (*p* = 0.032), i.e., lower levels of family organisation and higher levels of control were related to higher levels of sibling distress (*p* < 0.001). Trend for higher parental wellbeing related to lower levels of youth distress (*p* = 0.063).
				*Parent questionnaires:* YOQ‐2.0; FES; SGWB	
Mazaheri et al. 2012 et al., USA; Not reported	Not reported	100%	Caregiver quality of life, and psychosocial adjustment of siblings compared to general inpatient and outpatient samples of children with complex health conditions.	Mixed methods‐ standardised questionnaires for wellbeing analysed using *t‐*tests and compared with a pre‐existing normative sample, and semi structured interviews	*Siblings:* Quantitative: Self‐reported PTSD was significantly different from a school‐based sample (*p* = 0.05) and from siblings of children with cancer (*p* = 0.01), 92% of siblings had moderate‐to‐severe symptoms of post‐traumatic stress reaction, significantly lower perceived QoL in the school functioning domain compared to healthy controls (*p* < 0.05). Qualitative: Negative outlook on life.
				*Sibling measures*: PedsQL child/teen report, UCLA PTSD Index, PWS interviews	*Parents:* Quantitative: Poorer perceived health related QoL (HRQol) (*p* < 0.01) and family functioning (*p* < 0.01) than parents of children with complex chronic health conditions residing at home, and elevated levels of psychological distress. Parents perceived the sibling's HRQoL to be significantly poorer compared to parents of healthy children (*p* < 0.01) and the siblings themselves with School Functioning (*p* < 0.05) and Psychosocial Health (*p* < 0.01) being significantly different. Qualitative: Parents reflected on sibling rivalry, and the caregiving duties performed by the sibling.
				*Measures for mothers*: BSI, PedsQL‐parent proxy, PWS interviews	
Rae‐Seebach 2008; USA; Not reported	Not reported	80% (inadequate rationale for using a mixed methods design to address the research question)	Impact of Prader‐Willi syndrome on siblings' psychosocial, emotional, and behavioural adjustment and health related quality of life, also siblings' perceptions of caregiving ability including the impact this had on their psychosocial adjustment. Results compared with a pre‐existing normative sample(s)	Mixed methods‐using standard measures questionnaires for wellbeing, behaviour, attachment and family functioning, analysed using *t‐*tests, correlations to compare sibling and parent measures, and semi‐structured interviews. Attachment theory framework.	*Siblings:* Quantitative: Self‐reported PTSD was significantly different from a school‐based sample (*p* = 0.05) and from siblings of children with cancer (*p* = 0.01), 92% of siblings had moderate‐to‐severe symptoms of post‐traumatic stress reaction, significantly lower perceived HRQoL (*p* = 0.05), negative perceptions of family environment, unresolved attachment was significantly related to perceived emotional (un)availability of parents (*p* = 0.000) and PTSD severity index (*p* = 0.008), no significant difference in internalising and externalising behaviour when compared with donor and non‐donor siblings of paediatric bone marrow patients. Qualitative: disruptive changes in the family related to the behaviours or needs of the sibling with Prader‐Willi syndrome.
				*Sibling measures*: PedsQL, LEAP, AAUAQ, RPSF/RPSM, BASC‐2: SRP, UCLA PTSD index, KFD‐R drawing task and interviews	*Parents:* Quantitative: Poorer perceived QoL (*p* = 0.05) than parents of children with complex chronic health conditions. Parents perceived the sibling's HRQoL to be significantly poorer compared to the siblings themselves with school, physical, emotional and social functioning being significantly different (all at *p* < 0.01). Mothers' psychological distress was significantly related to the sibling's unresolved quality of attachment (*p* = 0.009), increased arousal of PTSD symptom (*p* = 0.011) and internalising behaviour (*p* = 0.020). Qualitative: stress arising from caregiving responsibilities.
				*Parent measures:* BSI, BASC‐2: PRS, RDI, and interviews	
Allen 2011; England; 18 months	ESRC and PWSA (UK)	100%	Experience of family members on routine aspects of living with a child with Prader‐Willi, including food management practises.	Qualitative family case studies; grounded theory.	‘Keeping occupied’ as one of the key practises to manage behaviour of the sibling with Prader‐Willi syndrome.
					b Highly emotionally charged nature of relationships within the households
Sibling reports only					
French 2022; Canada; 2 months	Not reported	80% (inconsistency between quantitative and qualitative results inadequately addressed)	Comparative study on experience of siblings of people with Prader‐Willi syndrome and siblings of people with no disability to assess stress, burden, and personal growth.	Mixed methods‐ x4 online surveys for stress and burden using standardised measures (PSS, PTGI, ZBI) analysed using analysis of variance, and qualitative question included towards the end of these surveys.	Quantitative: Significant difference between the two groups of siblings (*p* = 0.003) arising from caregiver burden experienced by the siblings of people with Prader‐Willi syndrome. Non‐significant difference between the two groups for personal growth and perceived stress.
					Qualitative: Siblings of people with Prader‐Willi syndrome mentioned both positive and negative impact on self, greater sense of burden, and stress related to several factors, such as family and school matters.
O'Neill and Murray 2016; England, N/A	Not reported	100%	Depression and anxiety in siblings of people with developmental disabilities including Prader‐Willi syndrome compared to a normative control group of siblings.	Quantitative standardised questionnaire for depression and anxiety (HADS) analysed via analyses of variance, moderation analyses using correlation and multiple regression.	Siblings of individuals with ASD or Prader‐Willi syndrome had higher levels of anxiety (*p* = 0.002) than the control group. Siblings of individuals with Prader‐Willi syndrome also had a high association between anxiety and high levels of maternal education. Differences in depression were non‐significant but being younger than the sibling with disabilities did significantly predict depression.
Parents reports only					
Meade et al. 2020, Ireland; not reported	National Children's Hospital Foundation	80% (sampling strategy unclear in relation to the research question)	Quality of life and burden for person with Prader‐Willi syndrome and caregiver. Views of child with Prader‐Willi syndrome and sibling were sought by parent proxy.	Quantitative standardised questionnaire for quality of life (PedsQL‐parent proxy) analysed via analysis of variance, t‐tests and correlation.	Parents reported higher mean values for quality of life for siblings compared to that of the child with Prader‐Willi syndrome (a difference of 13 points).

Abbreviations: AAUAQ, the adolescent unresolved attachment questionnaire; BASC‐2: PRS, The behaviour assessment scale for children, 2nd edition: parent rating scale; BASC‐2: SRP, The behaviour assessment scale for children, 2nd edition: child rating scale; BSI, brief symptom inventory; CD‐RISC 10, Connor‐Davidson resilience scale‐10; CES‐D, center for epidemiological studies depression scale; CPSS, child PTSD symptom scale; FES, family environment scale; GSI, global severity index; HADS, hospital anxiety and depression scale; HQ‐CT, hyperphagia questionnaire for clinical trials; KFDR, kinetic family drawing‐revised; LEAP, Lum emotional availability of parents; PANAS‐C, positive and negative affect schedule for children; PedsQL parent proxy, paediatric quality of life inventory; PedsQL, paediatric quality of life inventory—child/teen report; PSDI, positive symptom distress index; PSS, perceived stress scale; PST, positive symptom total; PTGI, post traumatic growth invention; RDI, reaction to diagnosis interview; RPSF/RPSM, relationship with parent's scale; SGWB, scales of general wellbeing; UCLA PTSA Index for DSM IV, Child Version, University of California at Los Angeles Posttraumatic Stress Disorder Reaction Index for DSM‐5; YOQ‐2.0, youth outcome questionnaire; ZBI, Zarit burden interview.

### 5.2 Funding

Funding sources were reported for three out of the seven studies. These were: ESRC and PWSA‐UK (UK); and from the US: Children's National Hospital Foundation and Department of Health and Human Services.

### 5.3 Study Quality Assessment

All seven studies had a quality appraisal MMAT score of 80% or above. Selection bias was a potential source of bias. Participants had an over‐representation of females, a wide age range, and were largely from the Northern Hemisphere, such as the USA, England, and Ireland. Deployment of quantitative methods and use of bespoke or standardised self‐report questionnaires could be contributing to reporting bias. There is a potential presence of confounding factors due to reliance on either the parental or the sibling perspective.

### 5.4 Qualitative Findings

Three themes emerged from the four qualitative reports across four studies, namely Impact on the siblings, Family relations, and Ways of coping Supporting Information B.

#### 5.4.1 Theme 1: Impact on the Siblings

This theme addressed the impact on the sibling's wellbeing across three qualitative reports (Mazaheri et al. 2012; Rae‐Seebach 2008; French 2022). Siblings reported an overall negative impact on self and emotional fatigue due to the unpredictability of their brother or sister with Prader‐Willi syndrome, requiring them to always be prepared for unexpected situations (Mazaheri et al. 2012), and resulting in unhappiness (Rae‐Seebach 2008). They shared feelings of despair and loneliness, and not being understood (Mazaheri et al. 2012). Siblings worried about upsetting their parents by making demands on their time, fearing they might be perceived as having negative feelings towards the sibling with Prader‐Willi syndrome (Mazaheri et al. 2012). There was some mention of developing resilience because of the family's situation (Rae‐Seebach 2008). There was an acknowledgement of the caregiver role that the siblings found themselves in either as an act of love or as an extra responsibility or parentification (Rae‐Seebach 2008; French 2022). Parental perceptions were contrasting with a mention of sibling rivalry and an acknowledgement of the sibling's caregiver role (Mazaheri et al. 2012).

#### 5.4.2 Theme 2: Family Relations

This theme explored the impact on relationships within the family and with people outside the family. Siblings often felt unable to communicate openly with their parents (Rae‐Seebach 2008). Feelings of ambivalence were common, possibly due to the unpredictable or perceived mean behaviour of their sibling with Prader‐Willi syndrome (Mazaheri et al. 2012). The complexity of emotions extended to empathy or lack thereof with people outside the family, including others with disabilities (French 2022). Family interactions were emotionally charged and prone to arguments (Allen 2011). The family environment was negatively impacted, with disruptions to routines and an inability to enjoy joint activities like parties and eating out (Rae‐Seebach 2008). Siblings felt that their brother or sister with Prader‐Willi syndrome occupied a central place in the family, demanding much of the parents' time (Mazaheri et al. 2012).

#### 5.4.3 Theme 3: Ways of Coping

This theme, which was covered in two reports focused on the coping strategies adopted by the siblings and had relatively fewer codes. Siblings managed their brother or sister's behaviour by keeping them occupied with puzzles or jigsaws (Allen 2011), seeking help from parents or external sources like caregivers and professional counselling (Mazaheri et al. 2012). Keeping the young person with Prader‐Willi syndrome engaged in activities was a means of controlling their behaviour and hunger (Allen 2011). Brief periods of respite, during which the sibling with Prader‐Willi syndrome was away from home, were found to help the siblings cope with their situation (Mazaheri et al. 2012).

### 5.5 Quantitative Findings

All six quantitative reports examined psychological factors. The results indicated that siblings exhibited significant PTSD symptoms (Bennett Murphy et al. 2022), anxiety (*p* = 0.002), and caregiver burden (French 2022). Parental reports on siblings highlighted significant psychological distress (Mazaheri et al. 2012). No significant differences were found in internalising or externalising behaviours compared to a normative sample (Rae‐Seebach 2008), nor in personal growth or perceived stress when compared to a control group (French 2022). Depression was not significant, but being younger than the sibling with disabilities significantly predicted depression (O'Neill and Murray 2016). A high association was found between anxiety and high levels of maternal education (O'Neill and Murray 2016). Parental perceptions of sibling quality of life (QoL) varied, with some reports indicating a lower perceived QoL compared to healthy controls (Mazaheri et al. 2012), whilst others showed a higher perceived QoL compared to the child with Prader‐Willi syndrome (Meade et al. 2020). Parental perception of sibling QoL was lower than the siblings' own perceptions (Mazaheri et al. 2012). There was a trend for higher parental well‐being related to lower levels of youth distress (*p* = 0.063) (Bennett Murphy et al. 2022) (Table 3).

Two of the six quantitative reports (Rae‐Seebach 2008; Bennett Murphy et al. 2022) covered family factors. Siblings perceived the family environment as negative. Attachment and distress were related, with the sibling's unresolved attachment significantly linked to perceived emotional (un)availability of parents and the severity of PTSD. Mothers' psychological distress was significantly related to the sibling's unresolved quality of attachment (*p* = 0.009), increased arousal of PTSD symptoms (*p* = 0.011), and internalising behaviour (*p* = 0.020) (Rae‐Seebach 2008). Lower levels of family organisation (*p* = 0.013) and higher levels of control (*p* = 0.032) were significantly related to higher levels of sibling distress (Bennett Murphy et al. 2022) (Table 3).

### 5.6 Narrative Synthesis

**FIGURE 2:**
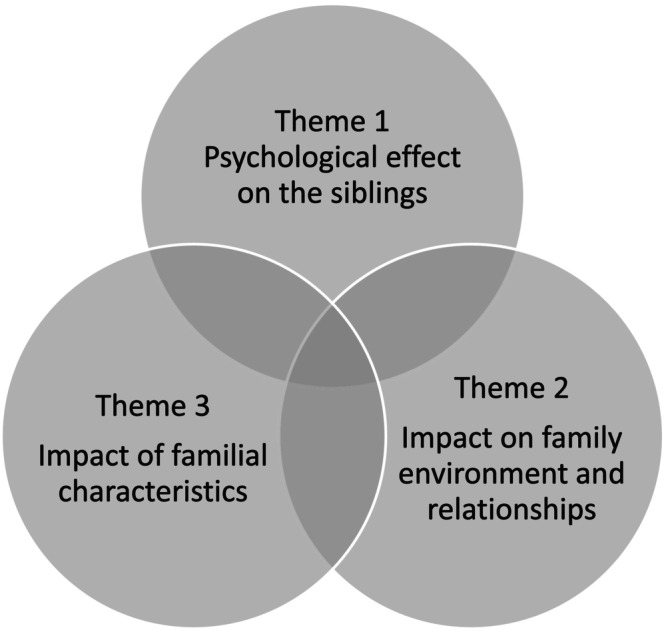
Narrative synthesis.

Consolidating the quantitative and qualitative reports using narrative synthesis resulted in three themes, namely, Psychological effect on the siblings, Impact on family environment and relationships, and Impact of familial characteristics (Figure 2).

#### 5.6.1 Theme 1: Psychological Effect on the Siblings

This theme highlights the psychological symptoms reported from the sibling perspective or by another family member, primarily the mother, in six studies (Bennett Murphy et al. 2022; Meade et al. 2020; O'Neill and Murray 2016; French 2022; Mazaheri et al. 2012; Rae‐Seebach 2008) with a focus on quantitative data. Overall, the psychological effects were predominantly negative. There was a discrepancy between the perceptions of siblings and parents. Parents rated the sibling's quality of life (Rae‐Seebach 2008; Mazaheri et al. 2012) lower than the siblings themselves. Positive aspects, such as coping strategies, were covered in qualitative reports. Siblings managed by finding practical solutions for handling the behaviour of their brother or sister with Prader‐Willi syndrome, as mentioned in the Allen (2011) study. Seeking advice and support from parents or professionals also helped them cope (Mazaheri et al. 2012).

#### 5.6.2 Theme 2: Impact on Family Environment and Relationships

This theme encompasses findings from three mixed methods studies (Mazaheri et al. 2012; Rae‐Seebach 2008; French 2022) and one qualitative study (Allen 2011), highlighting the impact on family dynamics and relationships. Siblings perceived the family environment negatively, noting disruptions to routines and a focus on the family member with Prader‐Willi syndrome. Their reflections on their caregiving role varied from affection to acknowledgment of parentification (Rae‐Seebach 2008; French 2022). Parents viewed the siblings' caregiving as a natural part of the family environment, alongside sibling rivalry (Mazaheri et al. 2012). Contrasting levels of organisation and control within the family environment were linked to sibling distress (Bennett Murphy et al. 2022). Family relationships were seen as disruptive and highly emotional (Allen 2011), with issues related to attachment and the emotional availability of parents. These experiences influenced siblings' interactions with people outside the family and their views on disability (French 2022).

#### 5.6.3 Theme 3: Impact of Familial Characteristics

This theme emerged across three studies (O'Neill and Murray 2016; Bennett Murphy et al. 2022; Rae‐Seebach 2008) and focused on the impact of the characteristics of the sibling with Prader‐Willi syndrome and the parent/mother on the other sibling. Certain factors were identified as risks for the sibling, such as having an older sibling with Prader‐Willi syndrome and high levels of maternal education and distress (O'Neill and Murray 2016; Rae‐Seebach 2008). Conversely, higher parental wellbeing was considered a protective factor (Bennett Murphy et al. 2022).

## 6 Discussion

This integrative review aimed to understand the experiences and support needs of siblings of individuals with Prader‐Willi syndrome from both the siblings' and the parents'/carers' perspectives. The integrative review design was chosen to gain a deeper understanding of the context and factors influencing the siblings' experiences through two review questions.

Regarding the first review question on sibling experience in Prader‐Willi syndrome, there was a notable tendency towards negative psychological experiences, such as stress and burden. Sibling experiences were complex, with concerns about adding to their parents' burden and mixed feelings towards their sibling with Prader‐Willi syndrome. This aligns with Goudie et al. (2013), who reported caregiver burden and mental health difficulties in siblings of individuals with various learning disabilities. The negative impact appears to be exacerbated by behavioural problems exhibited by the family member with additional needs: the greater the intensity of behavioural problems, the greater the negative effect on the sibling (Lardieri et al. 2000). Behavioural problems are also known to affect sibling relationships both in the presence and absence of a learning disability (Buist et al. 2013; Hastings and Petalas 2014; Petalas et al. 2012). Dietary or food management practises and associated caregiver burden were the focus of two studies (Allen 2011; Meade et al. 2020). Hyperphagia is associated with increased caregiver burden in families caring for a child with Prader‐Willi syndrome (Currie et al. 2024). Families living with and caring for someone with Prader‐Willi syndrome report extreme practises around food security and mealtimes (Currie et al. 2024). Since food and mealtimes are central to family life, hyperphagia can affect family practises and routines (Goldberg et al. 2002), potentially resulting in negative attitudes towards food in the sibling. However, focusing mainly on hyperphagia can mean that other aspects of the family's experiences are not well understood. Therefore, it is essential to consider the broader impact on the sibling within the family and other environments. The sibling experience is shaped by the family context, including the family environment, relationships, and familial characteristics. Similar to psychological experiences, family environment and relationships were negatively impacted. Siblings viewed the focus on their brother or sister with Prader‐Willi syndrome as negatively affecting their family life. This negative impact influenced their reactions to disabilities in general and compounded their experiences outside the family home, where they felt lonely and misunderstood. From this perspective, the sibling experience in Prader‐Willi syndrome appears to be characterised by loneliness both inside and outside the family home.

Sibling relational quality and health is known to be influenced by the activity levels and the presence or absence of an occupation or vocation in the sibling with the disability, rather than solely by the disability itself (Taylor and Hodapp 2012). As seen from this review, the familial characteristics also played a role; if the person with Prader‐Willi syndrome was sufficiently active and younger than the sibling, it fostered joint enjoyment, affection, and a stronger sibling bond. Parentification, as seen in this review, where older siblings or siblings take on a caregiving role for their family member with a disability (McHale et al. 2013), can negatively impact the sibling's well‐being (Hanöz et al. 2023). Individuals with Prader‐Willi syndrome often engage in activities obsessively. Families use this trait to keep them engaged as a behavioural management practise and to regulate their food intake (Allen 2011). However, this can be detrimental to sibling and family relationships. Families face the challenge of balancing behavioural and food demands, managing obsessions, and nurturing relationship quality. Higher levels of maternal education have been suggested to relate to supportive parenting and can act as a protective factor against child disruptive behaviour (Morawska et al. 2009). However, evidence from studies on autism and Down syndrome is inconclusive regarding parental education (Pollard et al. 2012) and needs further examination in Prader‐Willi syndrome. Findings from this review align with existing evidence on the relationship between maternal distress and behaviour problems in siblings (Williams et al. 2022). It is noteworthy that positive aspects of the sibling experience had limited coverage in the included studies. Overall, the sibling experience in Prader‐Willi syndrome is shaped by a complex interplay of several familial variables rather than the syndrome itself. Investigating the family context through an ecological systems lens (Bronfenbrenner 1979; Saxena and Adamsons 2013) might illustrate the causal nature of the psychological impact on the sibling.

Evidence was equivocal in relation to the second review question regarding parental perceptions of the sibling experience. Findings showed a reliance on either the sibling perspective *or* the parental perspective. There were limited joined‐up perspectives from the siblings with their own parent(s), which showed that parents might incorrectly understand the impact. Findings concur with research on parents of youth with autism (Rankin et al. 2017) and of children with Williams syndrome (Cebula et al. 2019). It is important to consider the perspectives of siblings with their own parent(s) as every sibling's experience is distinct, shaped by family dynamics, available support systems, and their individual personalities (Linimayr et al. 2025). Taking a multi‐source view and capturing the family's perceptions as a *family system* is important to support the family's wellbeing (Bennett Murphy et al. 2022). This could have implications for the feelings of agency experienced by the person with Prader‐Willi syndrome.

### 6.1 Methodology

Most of the studies included used quantitative scales to assess the extent of negative symptomatology with very limited evidence on positive aspects. Without including a qualitative aspect, it was difficult to ascertain *why* the siblings scored poorly on some of these scales. The voice of the unaffected siblings was missing, which could have allowed the positive aspects of sibling life to come to the fore, such as positive memories and a sense of belonging. This review indicated an evidence gap of holistic perspective from the sibling and family experiences in unison. As research into the experiences of siblings of people with Prader‐Willi syndrome is evolving, an exploratory qualitative design may be beneficial for generating future research directions. There is a lack of representation of males or about male relations, such as fathers and brothers. As caregiving experiences and wellbeing differ across genders (Marquis et al. 2019), evidence from this review is limited towards the female population. Although Prader‐Willi syndrome is equally represented across ethnicities, there was a lack of representation from the global south in the studies. Thus, our findings are limited to high‐income countries and might not transfer or generalise globally.

### 6.2 Theoretical Standpoint

The majority of the studies included in this review did not explicitly report their theoretical orientation. This contrasts with studies concerning the broader topic about supporting siblings of people with developmental disabilities, which have tended to use the family systems and the bioecological theoretical approach (e.g., Hastings 2014; Hastings et al. 2020; and Saxena and Adamsons 2013). For a detailed coverage of theories see (Hayden and Hastings 2022). A family‐centred approach has also been more prevalently assumed in other studies of family life, such as studies of acute paediatrics settings (e.g., Pettoello‐Mantovani et al. 2009; and Harrison 2010). These theoretical standpoints therefore comprise a good ‘tried and tested’ fit to understand the complex interplay of factors that shape the siblings’ experiences and outcomes from a family and ecological system perspective. Further research and intervention development relating to siblings of individuals with Prader‐Willi syndrome would naturally benefit from grounding the research in a clearly articulated theoretical standpoint and methods development.

### 6.3 Potential Biases in Reviewed Studies

Self‐selection bias is inherent and unavoidable due to the voluntary nature of participation. This potentially means that individuals and families who could benefit from the findings of the studies might not themselves be represented within the study samples. Participants tended to be female, which might also relate to self‐selection bias. The studies included in the review were all in English, with samples from high income countries. These risks highlight the experiences of people from predominantly Western regions of the world, thus limiting any global generalisations. Some of the studies referred to Prader‐Willi syndrome in general categorical terms (as a type of ‘disability’), which risks its distinct nature being disregarded (e.g., the experience of hyperphagia), and could lead to biassed findings.

### 6.4 Strengths and Limitations of the Review Process

Study quality and reliability checks, along with the use of multiple databases, mean less risk of bias in the results. Excluding publications which were not in the English language means that some potentially relevant studies, written in other languages, may have been missed. The updated round of search did not dramatically change the final number of included studies and thus provides increased confidence in the systematic search strategy. This might indicate that capturing the sibling *and* parent perspective is still an area of development in the field of Prader‐Willi syndrome research.

### 6.5 Conclusions

Findings from this important review in Prader‐Willi syndrome indicate that experiences of siblings are shaped by several familial variables and not simply by the presence of a family member with Prader‐Willi syndrome. This review highlights the need for representation of family as an integrated unit in Prader‐Willi syndrome research. Findings foreground the need for a family‐centred approach in future research and practise, from the early stages of disclosing the diagnosis to the family through to developing interventions for the siblings. Future research might therefore benefit from seeking multiple perspectives from varied angles, including from the person with Prader‐Willi syndrome and their parents. These factors might help to tailor interventions, which may be especially crucial when/if siblings take over the caregiving responsibility from their parents and to address the unmet support needs of siblings and families of people with Prader‐Willi syndrome.

#### 6.5.1 Implications for Research and Practise

Capturing the perspectives of both parents/carers *and* the siblings, examining familial characteristics, boosting recruitment of male participants, using an asset‐based approach (Emerson and Hatton 2014) to understand positive experiences, and developing exploratory qualitative research can pave the way for future rigorous studies and trials. Research can consider the views of the person with Prader‐Willi syndrome to get a wholistic picture of the impact on the family system and the impact it can have on the agency of the person with Prader‐Willi syndrome. Prader‐Willi syndrome has its set of unique associated challenges and therefore studies on genetic disabilities ‘in general’ risk detriment to what is known about the experience of being the sibling of a person with Prader‐Willi syndrome. It is important for policy makers and practitioners to consider the experiences and insights of family members when addressing the needs of the person with Prader‐Willi syndrome. Interventions to address negative effects, to consider individual characteristics of each family member, and to consider occupation and inclusion of people with Prader‐Willi syndrome might also be useful.

## Author Contributions

**Meghana Wadnerkar Kamble:** lead author, writing (final draft, review and editing), funding acquisition, formal analysis, data curation, conceptualisation. **Jen Dawe:** co‐lead author, writing (first draft, review and editing), formal analysis, data curation, conceptualisation. **Karen Bunning:** writing (review and editing), formal analysis, conceptualisation.

## Funding

This work was supported by Prader‐Willi Syndrome Association UK, R211454. Funding sources were reported for three out of the seven studies. These were: ESRC and PWSA‐UK (UK); and from the US: Children's National Hospital Foundation and Department of Health and Human Services.

## Conflicts of Interest

The authors declare no conflicts of interest.

## Supporting information


**Data S1:** Supporting Information.

## Data Availability

Data sharing is not applicable to this article as no new data were created or analyzed in this study.
