# Supplementary material for: Experiences and Support Needs of Siblings of Individuals With Prader‐Willi Syndrome: An Integrative Systematic Review
Source: J Appl Res Intellect Disabil. 2026 Jan 11;39(1):e70171. doi: 10.1111/jar.70171 (PMC12791193; doi:10.1111/jar.70171)
Supplement: Supplementary file 1 — Data S1: Supporting Information. [file JAR-39-e70171-s001.docx]

**Supplemental information**

**Supplemental information A**

**Detailed search strategy**

**A.1 COCHRANE central register search strategy**

| **Set** | **Search string** |
| --- | --- |
| 1 | ((“Prader-Willi syndrome” OR PWS OR “prader-willi” OR “neurodevelopmental condition*” OR “developmental disabilit*”)):ti,ab,kw |
| 2 | (sibling* OR brother* OR sister* OR parent* OR carer* OR caregiver* OR grandparent* OR adopt* OR foster* OR local authorit* OR “siblings of children with disabilit*”) |
| 3 | (support* OR help* OR experience* OR perception* OR attitude* OR view* OR feeling* OR emotion* OR affect*) |
| 4 | #1 AND #2 AND #3 |

**A.2 PubMed search strategy**

| **Set** | **Search string** |
| --- | --- |
| 1 | ((("prader-willi syndrome"[tw] OR pws OR “prader#willi” OR "developmental disabilities"[MeSH Terms] OR "neurodevelopmental condition*"[tw]))) AND 2000:2022[dp] Filters: Publication date from 2000/10/01 to 2022/10/20 |
| 2 | (((support[tw] OR help[tw] OR experience[tw] OR perception[MeSH Terms] OR attitude[MeSH Terms] OR emotions)[MeSH Terms] OR view*[tw] OR feeling*[tw] OR emotion*[tw] OR affect*[tw]))) AND 2000:2022[dp] Filters: Publication date from 2000/10/01 to 2022/10/20 |
| 3 | (((siblings[MeSH Terms] OR parents[MeSH Terms] OR caregivers[MeSH Terms] OR grandparents[MeSH Terms] OR adopt*[tw] OR foster*[tw] OR local authorit*[tw] OR "siblings of children with disabilit*")[tw]))) AND 2000:2022[dp] Filters: Publication date from 2000/10/01 to 2022/10/20 |
| 4 | ((#1) AND #2) AND #3 Filters: Publication date from 2000/10/01 to 2022/10/20 |

**A.3 Embase search strategy**

| **Set** | **Search string** |
| --- | --- |
| 1 | (prader-willi syndrome or pws or prader-willi or neurodevelopmental conditions or developmental disabilit*).ab,ti |
| 2 | limit 1 to (human and english langauge and yr=2000 - 2022) |
| 3 | (sibling* or brother* or sister* or parent* or carer* or caregiver* or grandparent* or adopt* or foster* or local authorit* or siblings or children with disabilit*).ab.ti |
| 4 | limit 3 to (human and english language and yr="2000 - 2022") |
| 5 | (support* or help* or experience* or perception* or attitude* or view* or feeling* or emotion*  or affect*).ab.ti |
| 6 | limit 5 to (human and english language and yr="2000 - 2022") |
| 7 | 2 and 4 |
| 8 | 6 and 7 |

**A.4 CINAHL search strategy**

(“Prader-Willi syndrome” OR PWS OR “prader-willi” OR “neurodevelopmental condition*” OR “developmental disabilit*”) AND (sibling* OR brother* OR sister* OR parent* OR carer* OR caregiver* OR grandparent* OR adopt* OR foster* OR local authorit* OR “siblings of children with disabilit*”) AND (support* OR help* OR experience* OR perception* OR attitude* OR view* OR feeling* OR emotion* OR affect*)

**A.5 PsychINFO search strategy**

(“Prader-Willi syndrome” OR PWS OR “prader-willi” OR "prader#willi" “neurodevelopmental condition*” OR “developmental disabilit*”) AND (sibling* OR brother* OR sister* OR parent* OR carer* OR caregiver* OR grandparent* OR adopt* OR foster* OR local authorit* OR “siblings of children with disabilit*”) AND (support* OR help* OR experience* OR perception* OR attitude* OR view* OR feeling* OR emotion* OR affect*)

**A.6 Web of Science search strategy**

(AB=("prader-willi syndrome" OR pws OR "prader-willi" OR "neurodevelopmental condition*" OR "developmental disabilit*")) AND (AB=(sibling* OR brother* OR sister* OR parent* OR carer* OR caregiver* OR grandparent* OR adopt* OR foster* OR "local authorit*" OR "siblings of children with disabilit*")) AND (AB=(support* OR help* OR experience* OR perception* OR attitude* OR view* OR feeling* OR emotion* OR affect*))

**A.7 Scopus search strategy**

( TITLE-ABS-KEY ( ( "Prader-Willi syndrome"  OR  pws  OR  "prader-willi"  OR  "neurodevelopmental condition*"  OR  "developmental disabilit*" ) )  AND  TITLE-ABS-KEY ( ( sibling*  OR  brother*  OR  sister*  OR  parent*  OR  carer*  OR  caregiver*  OR  grandparent*  OR  adopt*  OR  foster*  OR  local  AND authorit*  OR  "siblings of children with disabilit*" ) )  AND  TITLE-ABS-KEY ( ( support*  OR  help*  OR  experience*  OR  perception*  OR  attitude*  OR  view*  OR  feeling*  OR  emotion*  OR  affect* ) ) )  AND  PUBYEAR  >  1999  AND  PUBYEAR  >  1999

**A.8 EBSCO search strategy**

AB ( (“Prader-Willi syndrome” OR PWS OR “prader-willi” OR “neurodevelopmental condition*" OR "developmental disabilit*") AND AB ( (sibling* OR brother* OR sister* OR parent* OR carer* OR caregiver* OR grandparent* OR adopt* OR foster* OR "local authorit*" OR "siblings of children with disabilit*") AND AB ( (support* OR help* OR experience* OR perception* OR attitude* OR view* OR feeling* OR emotion* OR affect

**Supplemental information B**

**Thematic process from Qualitative Reports**

| **Statements** | **Keywords** | **Codes** | **Themes** |
| --- | --- | --- | --- |
| (Mazaheri et al., 2013)  “Overall, my outlook on life is quite a bit worse compared with other people I know. I definitely don’t see myself as an optimist . . .. I feel like I have more serious things to think about and I worry a lot more about what can or could happen to people”  “It used to make me mad”  “..prepare myself for the inevitable blow-up”  “I can’t let my guard down”  “I never know when she’ll have a fit or blow up”  “I try to keep my feelings to myself”  “I’m worried about upsetting my parents”  Unhappiness, lack of spontaneity (Ree-Seebach, 2008).  Resilience (Ree-Seebach, 2008) | Worse outlook, worry, not an optimist, emotions  Never know, prepare  Keep feelings to self  Worried, upsetting  Unhappiness    Resilience | Code 1 Psychological effects | Theme 1 Impact on the siblings |
| “A lot of people can’t relate to my experience” (Mazaheri et al., 2013) | Others can’t relate | Code 2 Not being understood by others |  |
| More responsible for themselves (Ree-Seebach, 2008).  “I do sometimes feel as if (I’m) the parent” (French, 2022).  Responsibility (Ree-Seebach, 2008).  “Everything that I do for my sibling is done through love, I’ll never feel burden by it” (French, 2022). | Self-responsibility  I am the parent | Code 3 Caregiver role |  |
| Highly emotionally charged nature of relationships within the households (Allen, 2011).  Disruption in the family and disagreements relating to the needs of the brother or sister with Prader-Willi syndrome; lack of doing activities together, family disputes (Ree-Seebach, 2008).  Prader-Willi syndrome bossiness or meanness (Mazaheri et al., 2013)  “Everyone is busy with the child with Prader-Willi syndrome. They care but have more pressing issues..” (Mazaheri et al., 2013) | Emotionally charged, relationships  Disruption in the family  Meanness  Everyone is busy | Code 1 Relating to family | Theme 2 Family relations |
| (French, 2022)  “I believe that  having siblings has helped develop my social skills, empathy, and understanding of others…”  “I am less compassionate to people with disabilities” | Develop, skills  Less compassionate | Code 2 Relating to others |  |
| (Mazaheri et al., 2013)  “My daughter is jealous of the child with Prader-Willi syndrome because she is beautiful. I think sometimes she feels embarrassed by the child with Prader-Willi syndrome”  “He helps by making sure that the child with Prader-Willi syndrome sticks to his diet, being responsible for himself, cleaning up and doing his chores. He feels bad that the child with Prader-Willi syndrome has to deal with Prader-Willi syndrome.” | Jealousy  Help, responsible for self | Code 3 Parental perceptions |  |
| “She (sister with PWS, 1 year age difference) has her puzzle books and she does them a lot. I’m not allowed (by sister with Prader-Willi syndrome) to do them, but I don’t mind really…” (Allen, 2011).  “If there were no caregivers or if my sister with Prader-Willi syndrome wasn’t gone for most weekends, it would be a lot harder” (Mazaheri et al., 2013)  When I do talk, I talk to my mom or my counsellor because I want advice (Mazaheri et al., 2013) | Keeping occupied  Respite  Sharing | Code 1 Behaviour management | Theme 3 Ways of coping |
